# 8-Hydr­oxy-5,6,7-trimeth­oxy-2-phenyl-4*H*-chromen-4-one

**DOI:** 10.1107/S1600536808010696

**Published:** 2008-04-23

**Authors:** Jahyr E. Theodoro, Djalma Santos, Hiram Pérez, Maria Fátima das Graças Fernandes da Silva, J. Ellena

**Affiliations:** a Instituto de Física de São Carlos-USP, Cx Postal 369, 13560-970 São Carlos, SP, Brazil; b Departamento Química-UFSCar, Cx Postal 676, 13565-905 São Carlos, SP, Brazil; c Departamento de Química Inorgánica, Facultad de Química, Universidad de la Habana, Habana 10400, Cuba

## Abstract

In the title compound, C_18_H_16_O_6_, the benzopyran group is essentially planar, with the O atoms of the substituent groups lying close to its mean plane. The mol­ecular conformation is governed by intra­molecular inter­actions. The crystal packing is mainly determined by one classical inter­molecular hydrogen bond which gives rise to the formation of an infinite chain along the *a* axis.

## Related literature

For related literature, see: Chebib & Johnston (2000 ▶); Medina *et al.* (1998 ▶).
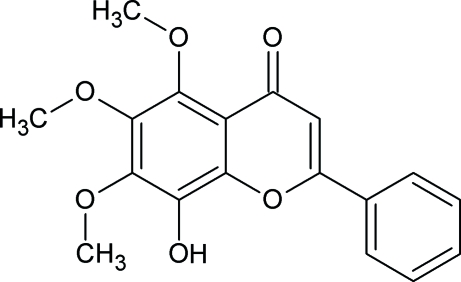


## Experimental

### 

#### Crystal data

C_18_H_16_O_6_
*M* *_r_* = 328.32
Triclinic, 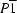

*a* = 8.4536 (2) Å
*b* = 9.0878 (2) Å
*c* = 10.7832 (3) Å
α = 79.545 (2)°
β = 71.5640 (10)°
γ = 86.925 (2)°
*V* = 772.85 (3) Å^3^
*Z* = 2
Mo *K*α radiation
μ = 0.11 mm^−1^
*T* = 294 K
0.22 × 0.19 × 0.11 mm

#### Data collection

Nonius Kappa CCD diffractometer
Absorption correction: none
20499 measured reflections
3153 independent reflections
2512 reflections with *I* > 2σ(*I*)
*R*_int_ = 0.049

#### Refinement

*R*[*F* ^2^ > 2σ(*F* ^2^)] = 0.042
*wR*(*F* ^2^) = 0.123
*S* = 1.07
3153 reflections
218 parameters
H-atom parameters constrained
Δρ_max_ = 0.25 e Å^−3^
Δρ_min_ = −0.24 e Å^−3^

### 

Data collection: *COLLECT* (Nonius, 2000 ▶); cell refinement: *COLLECT*; data reduction: *DENZO* and *SCALEPACK* (Otwinowski & Minor, 1997 ▶); program(s) used to solve structure: *SHELXS97* (Sheldrick, 2008 ▶); program(s) used to refine structure: *SHELXL97* (Sheldrick, 2008 ▶); molecular graphics: *ORTEP-3 for Windows* (Farrugia, 1997 ▶); software used to prepare material for publication: *WinGX* (Farrugia, 1999 ▶).

## Supplementary Material

Crystal structure: contains datablocks global, I. DOI: 10.1107/S1600536808010696/bg2176sup1.cif

Structure factors: contains datablocks I. DOI: 10.1107/S1600536808010696/bg2176Isup2.hkl

Additional supplementary materials:  crystallographic information; 3D view; checkCIF report

## Figures and Tables

**Table 1:** Hydrogen-bond geometry (Å, °)

*D*—H⋯*A*	*D*—H	H⋯*A*	*D*⋯*A*	*D*—H⋯*A*
O6—H8⋯O5	0.82	2.35	2.770 (1)	113
O6—H8⋯O2^i^	0.82	1.94	2.727 (1)	160

Symmetry code: (i) 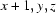.

**Fig. 1.**
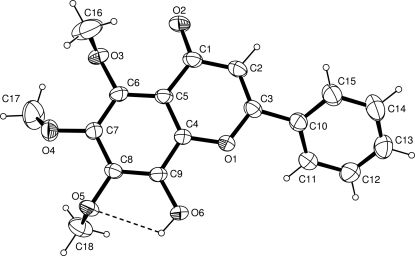
View of (I) (50% probability displacement ellipsoids)

**Fig. 2.**
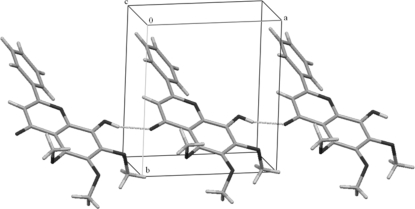
View of the intermolecular interaction that gives rise to the formation of a chain along the a axis.

## supplementary crystallographic information

### Comment

A number of differents flavones are known to have interesting modulatory activities at Gamma-aminobutyric acid receptors (GABA-A), an inhibitory neurotransmitter found in the nervous systems of widely divergent species. It is the main inhibitory neurotransmitter in the central nervous system (Medina *et al.*, 1998; Chebib & Johnston, 2000).

Figure 1 shows an *ORTEP* view of thr title compound, 8-hydroxy-5,6,7-trymethoxy-2-phenylchromen-4-one (I) with atom labeling and 50% probability displacement ellipsoids. The benzopyran group in (I) is essentially planar, with the oxygen atoms of the substituent groups lying close to its mean plane. The ring forms angles of 113.8 (4)°, 117.8 (3)° and 114.4 (2)° with the O3—C16, O4—C17 and O5—C18 methoxy groups, respectively, and 34.61 (4)° with the phenyl ring.

The molecular conformation is fixed by intramolecular interactions (Table 1 and Figure 1). The crystal packing is mainly determined by one classical intermolecular H bond which gives rise to the formation of an infinite chain along the *a* axis (Table 1 and Figure 2).

### Experimental

Selected parts of the *Z*. montana plant (Branches and leaves) were dried carefully by forced air at 40 °C and reduced to powder. The resulting material was macerated three times with hexane, followed with methanol at room temperature for 72 h each. After the evaporation of the solvent under reduced pressure, crude extracts were obtained. A well shaped single-crystal of the title compound was selected for the XRD experiments.

### Refinement

All the hydrogen atoms were stereochemically positioned and refined with a riding model. Hydrogen atoms of the CH and CH2 groups were set isotropic with a thermal parameter 20% greater than the equivalent isotropic displacement parameter of the atom to which each one was bonded. This percentage was set to 50% for the hydrogen atoms of the CH3 and OH groups.

### Crystal data

**Table:**

C_18_H_16_O_6_	*Z* = 2
*M**_r_* = 328.32	*F*_000_ = 344
Triclinic, *P*1	*D*_x_ = 1.411 Mg m^−^^3^
Hall symbol: -P 1	Mo *K*α radiation λ = 0.71073 Å
*a* = 8.4536 (2) Å	Cell parameters from 20513 reflections
*b* = 9.0878 (2) Å	θ = 2.9–26.4º
*c* = 10.7832 (3) Å	µ = 0.11 mm^−^^1^
α = 79.545 (2)º	*T* = 294 K
β = 71.5640 (10)º	Prism, yellow
γ = 86.925 (2)º	0.22 × 0.19 × 0.11 mm
*V* = 772.85 (3) Å^3^	

### Data collection

**Table:**

KappaCCD diffractometer	*R*_int_ = 0.049
φ scans and ω scans winth κ offsets	θ_max_ = 26.4º
Absorption correction: none	θ_min_ = 3.3º
20499 measured reflections	*h* = −10→10
3153 independent reflections	*k* = −11→11
2512 reflections with *I* > 2σ(*I*)	*l* = −13→13

### Refinement

**Table:**

Refinement on *F*^2^	H-atom parameters constrained
Least-squares matrix: full	*w* = 1/[σ^2^(*F*_o_^2^) + (0.0678*P*)^2^ + 0.1467*P*] where *P* = (*F*_o_^2^ + 2*F*_c_^2^)/3
*R*[*F*^2^ > 2σ(*F*^2^)] = 0.042	(Δ/σ)_max_ < 0.001
*wR*(*F*^2^) = 0.124	Δρ_max_ = 0.25 e Å^−^^3^
*S* = 1.07	Δρ_min_ = −0.24 e Å^−^^3^
3153 reflections	Extinction correction: SHELXL97 (Sheldrick, 2008), Fc^*^=kFc[1+0.001xFc^2^λ^3^/sin(2θ)]^-1/4^
218 parameters	Extinction coefficient: 0.043 (11)

### Special details

**Table:**

Geometry. All e.s.d.'s (except the e.s.d. in the dihedral angle between two l.s. planes) are estimated using the full covariance matrix. The cell e.s.d.'s are taken into account individually in the estimation of e.s.d.'s in distances, angles and torsion angles; correlations between e.s.d.'s in cell parameters are only used when they are defined by crystal symmetry. An approximate (isotropic) treatment of cell e.s.d.'s is used for estimating e.s.d.'s involving l.s. planes.

### Fractional atomic coordinates and isotropic or equivalent isotropic displacement parameters (Å^2^)

**Table:**

	*x*	*y*	*z*	*U*_iso_*/*U*_eq_	
O1	0.40508 (11)	0.55938 (11)	0.19334 (9)	0.0343 (3)	
O2	0.05483 (13)	0.68927 (14)	0.00920 (12)	0.0550 (3)	
O3	0.29650 (12)	0.85689 (12)	−0.19401 (10)	0.0428 (3)	
O4	0.63074 (13)	0.94104 (12)	−0.28518 (10)	0.0441 (3)	
O5	0.83892 (11)	0.83118 (11)	−0.14212 (9)	0.0362 (3)	
O6	0.71943 (11)	0.63816 (12)	0.09768 (9)	0.0395 (3)	
C1	0.16504 (16)	0.64776 (16)	0.06016 (15)	0.0350 (3)	
C2	0.13054 (16)	0.54641 (16)	0.18467 (14)	0.0368 (3)	
C3	0.24520 (16)	0.50867 (15)	0.24709 (13)	0.0330 (3)	
C4	0.45089 (16)	0.65179 (15)	0.07187 (13)	0.0 (3)	
C5	0.33896 (15)	0.70155 (15)	0.00135 (13)	0.0301 (3)	
C6	0.39952 (16)	0.80083 (15)	−0.12037 (13)	0.0320 (3)	
C7	0.56421 (16)	0.84828 (15)	−0.16628 (13)	0.0313 (3)	
C8	0.67300 (15)	0.79300 (15)	−0.09404 (13)	0.0296 (3)	
C9	0.61900 (15)	0.69429 (15)	0.02441 (13)	0.0295 (3)	
C10	0.21737 (17)	0.41557 (16)	0.37915 (13)	0.0353 (3)	
C11	0.30333 (19)	0.44556 (19)	0.46281 (16)	0.0459 (4)	
C12	0.2755 (2)	0.3598 (2)	0.58706 (16)	0.0547 (5)	
C13	0.1650 (2)	0.2423 (2)	0.62864 (17)	0.0552 (5)	
C14	0.0795 (2)	0.2109 (2)	0.54662 (17)	0.0573 (5)	
C15	0.1043 (2)	0.29793 (19)	0.42257 (16)	0.0469 (4)	
C16	0.2848 (3)	0.7646 (3)	−0.2840 (2)	0.0738 (6)	
C17	0.5601 (3)	1.0840 (2)	−0.3033 (2)	0.0708 (6)	
C18	0.8774 (2)	0.96476 (19)	−0.10671 (19)	0.0514 (4)	
H8	0.8139	0.672	0.0606	0.059*	
H8A	0.0242	0.5052	0.2237	0.044*	
H17	0.3799	0.5239	0.4346	0.055*	
H18	0.3319	0.3816	0.6429	0.066*	
H19	0.1477	0.1839	0.7121	0.066*	
H20	0.0049	0.131	0.5748	0.069*	
H21	0.045	0.2774	0.3682	0.056*	
H22A	0.9951	0.9838	−0.1435	0.077*	
H22B	0.8451	0.9539	−0.0118	0.077*	
H22C	0.818	1.0469	−0.1409	0.077*	
H27A	0.2116	0.8102	−0.3321	0.111*	
H27B	0.2415	0.6682	−0.2355	0.111*	
H27C	0.3934	0.753	−0.3452	0.111*	
H30A	0.6176	1.1374	−0.3902	0.106*	
H30B	0.5701	1.1381	−0.2375	0.106*	
H30C	0.4444	1.074	−0.2946	0.106*	

### Atomic displacement parameters (Å^2^)

**Table:**

	*U*^11^	*U*^22^	*U*^33^	*U*^12^	*U*^13^	*U*^23^
O1	0.0240 (5)	0.0430 (6)	0.0331 (5)	−0.0036 (4)	−0.0093 (4)	0.0024 (4)
O2	0.0248 (5)	0.0739 (8)	0.0630 (7)	−0.0045 (5)	−0.0224 (5)	0.0132 (6)
O3	0.0334 (5)	0.0540 (6)	0.0446 (6)	0.0043 (5)	−0.0228 (5)	0.0007 (5)
O4	0.0364 (6)	0.0472 (6)	0.0381 (6)	0.0029 (4)	−0.0061 (4)	0.0083 (5)
O5	0.0218 (5)	0.0456 (6)	0.0403 (5)	−0.0040 (4)	−0.0082 (4)	−0.0067 (4)
O6	0.0240 (5)	0.0570 (6)	0.0364 (5)	−0.0039 (4)	−0.0151 (4)	0.0057 (4)
C5	0.0227 (6)	0.0339 (7)	0.0350 (7)	0.0019 (5)	−0.0114 (5)	−0.0058 (6)
C9	0.0233 (6)	0.0362 (7)	0.0309 (7)	0.0014 (5)	−0.0117 (5)	−0.0051 (5)
C4	0.0239 (6)	0.0336 (7)	0.0302 (7)	0.0006 (5)	−0.0097 (5)	−0.0036 (5)
C8	0.0216 (6)	0.0345 (7)	0.0328 (7)	−0.0001 (5)	−0.0081 (5)	−0.0071 (5)
C1	0.0239 (6)	0.0391 (7)	0.0434 (8)	0.0009 (5)	−0.0134 (6)	−0.0057 (6)
C6	0.0264 (6)	0.0369 (7)	0.0354 (7)	0.0051 (5)	−0.0153 (6)	−0.0044 (6)
C2	0.0218 (6)	0.0427 (8)	0.0430 (8)	−0.0027 (5)	−0.0077 (6)	−0.0039 (6)
C7	0.0281 (7)	0.0336 (7)	0.0307 (7)	0.0020 (5)	−0.0093 (5)	−0.0024 (5)
C3	0.0247 (6)	0.0361 (7)	0.0355 (7)	−0.0012 (5)	−0.0057 (5)	−0.0057 (6)
C10	0.0278 (7)	0.0403 (8)	0.0332 (7)	0.0019 (6)	−0.0047 (5)	−0.0041 (6)
C11	0.0360 (8)	0.0560 (10)	0.0431 (8)	−0.0103 (7)	−0.0132 (7)	0.0023 (7)
C12	0.0441 (9)	0.0773 (12)	0.0404 (9)	−0.0070 (8)	−0.0160 (7)	0.0031 (8)
C13	0.0510 (10)	0.0644 (11)	0.0386 (9)	−0.0040 (8)	−0.0068 (7)	0.0091 (8)
C14	0.0588 (11)	0.0530 (10)	0.0482 (10)	−0.0184 (8)	−0.0038 (8)	0.0031 (8)
C15	0.0462 (9)	0.0517 (9)	0.0393 (8)	−0.0123 (7)	−0.0082 (7)	−0.0052 (7)
C16	0.0701 (13)	0.1072 (17)	0.0656 (12)	0.0106 (12)	−0.0457 (11)	−0.0277 (12)
C17	0.0703 (13)	0.0491 (10)	0.0732 (13)	0.0092 (9)	−0.0106 (10)	0.0160 (9)
C18	0.0378 (8)	0.0454 (9)	0.0718 (11)	−0.0098 (7)	−0.0173 (8)	−0.0089 (8)

### Geometric parameters (Å, °)

**Table:**

O3—C6	1.3753 (15)	C5—C6	1.4120 (19)
O3—C16	1.422 (2)	C5—C1	1.4757 (18)
O2—C1	1.2341 (16)	C9—C8	1.3798 (18)
O5—C8	1.3724 (15)	C9—C4	1.3992 (18)
O5—C18	1.4193 (19)	C8—C7	1.4024 (18)
C18—H22A	0.9600	C1—C2	1.438 (2)
C18—H22B	0.9600	C6—C7	1.3857 (19)
C18—H22C	0.9600	C2—C3	1.3430 (19)
C16—H27A	0.9600	C2—H8A	0.9300
C16—H27B	0.9600	C3—C10	1.4735 (19)
C16—H27C	0.9600	C10—C15	1.386 (2)
O6—C9	1.3552 (15)	C10—C11	1.393 (2)
O6—H8	0.8200	C15—C14	1.384 (2)
O4—C7	1.3687 (16)	C15—H21	0.9300
O4—C17	1.408 (2)	C12—C13	1.372 (3)
C17—H30A	0.9600	C12—C11	1.378 (2)
C17—H30B	0.9600	C12—H18	0.9300
C17—H30C	0.9600	C11—H17	0.9300
O1—C3	1.3599 (16)	C13—C14	1.380 (3)
O1—C4	1.3733 (16)	C13—H19	0.9300
C5—C4	1.4009 (17)	C14—H20	0.9300
			
C6—O3—C16	113.82 (13)	C9—C8—C7	121.45 (12)
C8—O5—C18	114.34 (11)	O2—C1—C2	121.77 (13)
O5—C18—H22A	109.5	O2—C1—C5	123.02 (13)
O5—C18—H22B	109.5	C2—C1—C5	115.18 (11)
H22A—C18—H22B	109.5	O3—C6—C7	118.28 (12)
O5—C18—H22C	109.5	O3—C6—C5	121.32 (12)
H22A—C18—H22C	109.5	C7—C6—C5	120.36 (12)
H22B—C18—H22C	109.5	C3—C2—C1	122.86 (12)
O3—C16—H27A	109.5	C3—C2—H8A	118.6
O3—C16—H27B	109.5	C1—C2—H8A	118.6
H27A—C16—H27B	109.5	O4—C7—C6	122.84 (12)
O3—C16—H27C	109.5	O4—C7—C8	117.18 (12)
H27A—C16—H27C	109.5	C6—C7—C8	119.84 (12)
H27B—C16—H27C	109.5	C2—C3—O1	121.83 (12)
C9—O6—H8	109.5	C2—C3—C10	126.44 (12)
C7—O4—C17	117.76 (13)	O1—C3—C10	111.69 (11)
O4—C17—H30A	109.5	C15—C10—C11	119.00 (14)
O4—C17—H30B	109.5	C15—C10—C3	120.60 (13)
H30A—C17—H30B	109.5	C11—C10—C3	120.40 (13)
O4—C17—H30C	109.5	C14—C15—C10	120.13 (15)
H30A—C17—H30C	109.5	C14—C15—H21	119.9
H30B—C17—H30C	109.5	C10—C15—H21	119.9
C3—O1—C4	119.21 (10)	C13—C12—C11	120.27 (16)
C4—C5—C6	117.91 (12)	C13—C12—H18	119.9
C4—C5—C1	117.68 (12)	C11—C12—H18	119.9
C6—C5—C1	124.41 (12)	C12—C11—C10	120.38 (15)
O6—C9—C8	123.53 (11)	C12—C11—H17	119.8
O6—C9—C4	118.48 (11)	C10—C11—H17	119.8
C8—C9—C4	117.98 (11)	C12—C13—C14	119.99 (15)
O1—C4—C9	114.47 (11)	C12—C13—H19	120.0
O1—C4—C5	123.14 (11)	C14—C13—H19	120.0
C9—C4—C5	122.39 (12)	C13—C14—C15	120.22 (16)
O5—C8—C9	117.81 (11)	C13—C14—H20	119.9
O5—C8—C7	120.68 (12)	C15—C14—H20	119.9
			
C3—O1—C4—C9	−178.49 (11)	C5—C1—C2—C3	3.3 (2)
C3—O1—C4—C5	2.30 (19)	C17—O4—C7—C6	60.8 (2)
O6—C9—C4—O1	2.27 (18)	C17—O4—C7—C8	−123.44 (17)
C8—C9—C4—O1	−176.89 (11)	O3—C6—C7—O4	−4.1 (2)
O6—C9—C4—C5	−178.52 (12)	C5—C6—C7—O4	178.13 (12)
C8—C9—C4—C5	2.3 (2)	O3—C6—C7—C8	−179.74 (12)
C6—C5—C4—O1	177.67 (12)	C5—C6—C7—C8	2.4 (2)
C1—C5—C4—O1	−1.7 (2)	O5—C8—C7—O4	−0.13 (19)
C6—C5—C4—C9	−1.5 (2)	C9—C8—C7—O4	−177.50 (12)
C1—C5—C4—C9	179.16 (12)	O5—C8—C7—C6	175.80 (12)
C18—O5—C8—C9	−94.47 (15)	C9—C8—C7—C6	−1.6 (2)
C18—O5—C8—C7	88.06 (16)	C1—C2—C3—O1	−2.9 (2)
O6—C9—C8—O5	2.7 (2)	C1—C2—C3—C10	174.87 (13)
C4—C9—C8—O5	−178.22 (11)	C4—O1—C3—C2	0.0 (2)
O6—C9—C8—C7	−179.89 (12)	C4—O1—C3—C10	−178.04 (11)
C4—C9—C8—C7	−0.8 (2)	C2—C3—C10—C15	34.3 (2)
C4—C5—C1—O2	176.96 (14)	O1—C3—C10—C15	−147.80 (14)
C6—C5—C1—O2	−2.4 (2)	C2—C3—C10—C11	−145.11 (16)
C4—C5—C1—C2	−0.99 (19)	O1—C3—C10—C11	32.83 (18)
C6—C5—C1—C2	179.69 (13)	C11—C10—C15—C14	−0.8 (2)
C16—O3—C6—C7	94.31 (17)	C3—C10—C15—C14	179.80 (15)
C16—O3—C6—C5	−87.89 (18)	C13—C12—C11—C10	1.2 (3)
C4—C5—C6—O3	−178.70 (12)	C15—C10—C11—C12	−0.3 (2)
C1—C5—C6—O3	0.6 (2)	C3—C10—C11—C12	179.07 (15)
C4—C5—C6—C7	−1.0 (2)	C11—C12—C13—C14	−0.9 (3)
C1—C5—C6—C7	178.37 (13)	C12—C13—C14—C15	−0.2 (3)
O2—C1—C2—C3	−174.72 (15)	C10—C15—C14—C13	1.1 (3)

### Hydrogen-bond geometry (Å, °)

**Table:**

*D*—H···*A*	*D*—H	H···*A*	*D*···*A*	*D*—H···*A*
O6—H8···O5	0.82	2.35	2.770 (1)	113
O6—H8···O2^i^	0.82	1.94	2.727 (1)	160

Symmetry codes: (i) *x*+1, *y*, *z*.
